# Arbuscular Mycorrhizal Community in Roots and Nitrogen Uptake Patterns of Understory Trees Beneath Ectomycorrhizal and Non-ectomycorrhizal Overstory Trees

**DOI:** 10.3389/fpls.2020.583585

**Published:** 2021-01-14

**Authors:** Chikae Tatsumi, Fujio Hyodo, Takeshi Taniguchi, Weiyu Shi, Keisuke Koba, Keitaro Fukushima, Sheng Du, Norikazu Yamanaka, Pamela Templer, Ryunosuke Tateno

**Affiliations:** ^1^Research Faculty of Agriculture, Hokkaido University, Sapporo, Japan; ^2^Graduate School of Agriculture, Kyoto University, Kyoto, Japan; ^3^Department of Biology, Boston University, Boston, MA, United States; ^4^Research Core for Interdisciplinary Sciences, Okayama University, Okayama, Japan; ^5^Arid Land Research Center, Tottori University, Tottori, Japan; ^6^School of Geographical Sciences, Southwest University, Chongqing, China; ^7^Center for Ecological Research, Kyoto University, Shiga, Japan; ^8^State Key Laboratory of Soil Erosion and Dryland Farming on Loess Plateau, Institute of Soil and Water Conservation, Chinese Academy of Sciences, Beijing, China; ^9^Field Science Education and Research Center, Kyoto University, Kyoto, Japan

**Keywords:** ^15^N natural abundance, arbuscular mycorrhizal fungi, dryland, ectomycorrhizal fungi (ECM fungi), mycorrhizal dependence, nitrate

## Abstract

Nitrogen (N) is an essential plant nutrient, and plants can take up N from several sources, including via mycorrhizal fungal associations. The N uptake patterns of understory plants may vary beneath different types of overstory trees, especially through the difference in their type of mycorrhizal association (arbuscular mycorrhizal, AM; or ectomycorrhizal, ECM), because soil mycorrhizal community and N availability differ beneath AM (non-ECM) and ECM overstory trees (e.g., relatively low nitrate content beneath ECM overstory trees). To test this hypothesis, we examined six co-existing AM-symbiotic understory tree species common beneath both AM-symbiotic black locust (non-ECM) and ECM-symbiotic oak trees of dryland forests in China. We measured AM fungal community composition of roots and natural abundance stable isotopic composition of N (δ^15^N) in plant leaves, roots, and soils. The root mycorrhizal community composition of understory trees did not significantly differ between beneath non-ECM and ECM overstory trees, although some OTUs more frequently appeared beneath non-ECM trees. Understory trees beneath non-ECM overstory trees had similar δ^15^N values in leaves and soil nitrate, suggesting that they took up most of their nitrogen as nitrate. Beneath ECM overstory trees, understory trees had consistently lower leaf than root δ^15^N, suggesting they depended on mycorrhizal fungi for N acquisition since mycorrhizal fungi transfer isotopically light N to host plants. Additionally, leaf N concentrations in the understory trees were lower beneath ECM than the non-ECM overstory trees. Our results show that, without large differences in root mycorrhizal community, the N uptake patterns of understory trees vary between beneath different overstory trees.

## Introduction

Soil nitrogen (N) is an essential nutrient for plants and it influences the productivity, composition, and functioning of forests (Tanner et al., 1998; LeBauer and Treseder, 2008; Wright et al., 2011). Plants primarily use soil inorganic N (ammonium and nitrate), but plants can also use organic N as a significant resource in low-N systems (Näsholm et al., 1998; Nordin et al., 2001; Jones and Kielland, 2002). In addition, plants can access various soluble organic compounds such as amino acids and peptides by associating with mycorrhizal fungi (Read and Perez-Moreno, 2003; Smith and Read, 2008; Pellitier and Zak, 2018). The differences in the mycorrhizal association among plants are also an important factor in regulating N uptake in host plants (Veresoglou et al., 2011; Mensah et al., 2015). Thus, plants have many strategies for taking up soil N and can change the form of N taken up in response to soil N availability, although each plant species has uptake preferences (Wei et al., 2015; Andersen et al., 2017; Uscola et al., 2017; Daryanto et al., 2019).

Overstory tree species can play an important role in N uptake of co-existing understory plants by changing the environmental conditions, including soil N availability and mycorrhizal inoculum source. The mycorrhizal type of the dominant tree species has come to be recognized as a potentially large factor controlling soil N availability (Phillips et al., 2013; Averill et al., 2014). Ectomycorrhizal (ECM) fungi can produce many hydrolytic or oxidative extracellular enzymes (Chalot and Brun, 1998; Courty et al., 2010; Averill and Finzi, 2011; Kohler et al., 2015), and obtain small organic N-bearing molecules from soil organic matter (SOM). As a result, ECM fungi are considered to limit the amount of N available for free-living microbes and slow soil decomposition and N transformations (Gadgil and Gadgil, 1971; Averill and Hawkes, 2016; Fernandez and Kennedy, 2016). On the other hand, arbuscular mycorrhizal (AM) fungi lack the ability to produce the extracellular enzymes and therefore are considered to have smaller effects than ECM fungi on soil N cycling (Read and Perez-Moreno, 2003; Smith and Read, 2008; Smith and Smith, 2011; Hodge and Storer, 2014). Accordingly, soil N availability, especially that of nitrate, is reported to be higher in AM than ECM forests (Phillips et al., 2013; Midgley and Phillips, 2016; Tatsumi et al., 2020). It is also possible that the higher nitrate content is caused by higher nitrification rates facilitated by lower acidity and higher ammonium pools in AM forests where decomposition rates are more rapid than ECM forests (Finzi et al., 1998; Phillips et al., 2013). Also, overstory trees associated with N-fixing bacteria can increase soil N availability (Rice et al., 2004; Von Holle et al., 2013), although it is not always the case (Wang et al., 2010).

It has been reported that some plants alter their N uptake patterns, such as slowing their rate of uptake and changing the N source that they take up, in response to changes in soil N availability, sometimes a result of N uptake by competitively superior plants (McKane et al., 2002; Ashton et al., 2010). Root mycorrhizal community also changes with soil N availability (Treseder and Allen, 2002; Porras-Alfaro et al., 2007) and with soil mycorrhizal inoculum sources in some cases (Duponnois et al., 2011; Bittebiere et al., 2019). On the other hand, even when the environmental conditions such as soil N availability changes, some plants do not change root mycorrhizal community composition (Martínez-García and Pugnaire, 2011; Gehring et al., 2017) and the primary source of N that they take up (Ashton et al., 2010; Wang and Macko, 2011). Also, differences in overstory trees may affect the root mycorrhizal community and N uptake pattern of understory trees. It has been reported that the understory tree community varies beneath AM and ECM overstory trees, and this is considered to be partly caused by differences in soil N availability (Jo et al., 2018).

Natural abundance stable isotopic composition of N (δ^15^N) can reveal plant N use processes (Koba et al., 2003; Templer et al., 2007). The δ^15^N in plant leaves and roots including mycorrhizal fungi can indicate plant dependence for N acquisition on mycorrhizal fungi, because mycorrhizal fungi transfer N with lower δ^15^N values to host plants (Kohzu et al., 2000; Emmerton et al., 2001; Hobbie and Colpaert, 2003); that is, mycorrhizal-dependent plants show leaf δ^15^N values that are lower than δ^15^N values of their roots including mycorrhizal fungi. Although the transfer of ^15^N-depleted N from AM fungi to host plants has not clearly been demonstrated compared to ECM fungi (Aguilar et al., 1998; Wheeler et al., 2000), some recent studies based on field observations, greenhouse experiments, and modeling support the idea that AM fungi slightly fractionate N (Schweiger, 2016; Jach-Smith and Jackson, 2020). The δ^15^N signature of leaves may also indicate the primary source of N that plants take up from soil (Takebayashi et al., 2010; Liu et al., 2018). For example, the δ^15^N of leaves tend to be low if plants depend on soil nitrate (Falkengren-grerup et al., 2004; Kahmen et al., 2008), because δ^15^N of soil nitrate is usually much lower than that of ammonium (Choi et al., 2005; Takebayashi et al., 2010). In addition, to determine if soil N availability is high, the measurement of δ^18^O of soil nitrate is useful because it can indicate whether the nitrate comes mostly from nitrification or atmospheric deposition, as δ^18^O values of nitrification-derived nitrate can be lower than 10‰ (Kendall et al., 2007; Fang et al., 2012). The δ^15^N values in the soils are also useful because net mineralization and nitrification rates are typically correlated positively with soil δ^15^N values (Templer et al., 2007).

Here, we examined root mycorrhizal community and δ^15^N and N concentrations of leaves and fine roots in co-existing understory plants, as well as the δ^15^N and soil N forms that plants can take up beneath non-ECM (black locust) and ECM (oak) overstory trees in dryland forests, to determine the effects of overstory trees on N acquisition by understory trees. In dryland forests, competition for N between ECM trees and free-living microbes is strong because N can be more limiting than water for plant production in dryland forests (Ren et al., 2017), which can result in a strong effect of the ECM trees on soil N availability. Black locust and oak are the two dominant species in our study region of dryland forests in China. The non-ECM species in this study (black locust) is associated with N-fixing bacteria, although the trees currently do not have active N-fixation as described below.

In this study, we hypothesized that root mycorrhizal community and N uptake patterns of understory trees vary beneath different overstory trees. We expected (1) the root mycorrhizal communities of understory trees to differ when occurring beneath different overstory trees in response to soil N availability and mycorrhizal inoculum source (derived from root mycorrhizal community in overstory trees), although the hypothesis about mycorrhizal inoculum source should be only in the case that overstory and understory trees are the same mycorrhizal type. We also expected (2) the understory trees beneath non-ECM overstory trees to be less dependent upon mycorrhizal fungi for N acquisition (i.e., leaf δ^15^N ≮ root δ^15^N) and instead to access highly available nitrate (i.e., leaf δ^15^N ≈ soil nitrate δ^15^N). On the other hand, we expected (3) the understory trees beneath ECM overstory trees to be highly dependent upon mycorrhizal fungi for N acquisition (i.e., leaf δ^15^N < root δ^15^N). We expected the understory tree species to be a stronger driver than the overstory tree species in determining N uptake patterns of understory trees.

## Materials and Methods

### Study Site

This study was conducted in a black locust (*Robinia pseudoacacia*) forest and an oak (*Quercus liaotungensis*) forest in the Loess Plateau of China. Black locust and oak are AM and ECM trees, respectively (Zhang et al., 2013; Yang et al., 2014). Black locust is also a tree with N-fixing symbionts, but there is no active N fixation at this site currently as evidenced by lack of root nodules in the roots and the lower abundance of N-fixing bacteria in soils of the black locust than oak forest (Supplementary Table 1). The study site is located near Yan’an city (Mt. Gonglushan, 36° 25 N, 109° 32 E) in Shaanxi Province, China. Black locust trees were widely planted in the 1960s and oak forests are the native climax forests, but most of the natural oak forests were already cut down by human activity (Lü et al., 2003). Thus, they are common forests in this region (Du et al., 2011), but it is difficult to find an oak forest, especially neighboring to a black locust forest. The vegetation type is categorized as a forest-steppe transitional zone (Yamanaka et al., 2014). Mean annual precipitation and mean annual air temperature are 514 mm and 10.2°C, respectively. This area experiences hot summers and cold winters, with heavy rainfall in the summer. According to Tateno et al. (2007), this area was colonized 65–75 years ago by people and was cultivated after native oak forests were cleared. Black locust was planted on abandoned cultivated lands around 45 years ago, and the oak forests comprised 65–70-year-old secondary forest.

Both sites have closed canopies, with more than 90% of the canopy occupied by the dominant species, black locust or oak. Six understory tree species appear commonly and frequently in both forests: *Rosa multiflora* (Rosaceae), *Cotoneaster multiflorus* (Rosaceae), *Platycladus orientalis* (Cupressaceae), *Syringa pekinensis* (Oleaceae), *Acer ginnala* (Sapindaceae), and *Acer stenolobum* (Sapindaceae). All of these species likely have AM associations based on recent plant surveys (Soudzilovskaia et al., 2020). These forests are located on a flat or gentle slope near the ridge at around 1,300 m elevation. The relative photosynthetic photon flux density (rPPFD) of the black locust forest and the oak forest are 7.6 ± 2.7 and 9.7 ± 3.2 (%), respectively.

### Sample Collection

We examined five mature individual trees (each 3–5 m height) for each understory tree species in each forest (ca. 50 m × 200 m); the five replications were a minimum of 20–30 m from each other. We also examined five mature individual overstory trees in each forest; the five overstory trees were a minimum of 20–30 m from each other. We sampled healthy leaves and fine roots from each individual. Approximately 10 g of root samples (mostly fine roots but including some coarse roots) were collected from surface soils (0–30 cm depth) where the majority of roots are located. To avoid contamination from the roots of other trees, all root systems were traced from the trunk of the selected trees. Root samples were separated into subsamples for isotopic analysis and DNA analysis. Approximately 10 g of leaf samples were collected randomly from each tree crown and the petiole was included. We also collected mineral soil from 0–10 cm depth around each tree individual to detect whether extractable N content is specifically determined by the tree individual (Supplementary Figure 1). Preliminary analyses showed that the effect of overstory tree type (ECM vs. non-ECM) on soil extractable N content was much larger than the effect of tree species, so we measured the δ^15^N only for each forest representative soils. Collection of leaf, root, and the soils around trees was conducted in mid-September 2015.

For the measurement of δ^15^N of the soil extractable N associated with the overstory tree species of each forest, additional mineral soil samples (0–10 cm depth) were collected from five locations beneath the overstory trees of each forest. The soil sampling locations were approximately 5 m from each other. These samples were collected in June and September of 2016. Each soil sample was divided into a subsample for water content analysis, and for N extraction to determine δ^15^N. In order to determine the average value over the growing season, soil inorganic N was captured by ion exchange resin capsules each containing about 1 g of mixed ion exchange resins (IER; PST-2, Unibest, Bozeman, MT, United States). Seven resin capsules were placed at a depth of 10 cm in soil beneath the overstory tree species of each forest beginning in June 2016. The resin capsules were *ca*. 10 m from one another. The resin capsules were collected after 3 months (in September 2016). The collected resin capsules were washed well with distilled water to remove adhering soils.

For additional measurements of δ^15^N in bulk soil, soil samples (0–10 cm depth and 20–30 cm depth) were collected at four locations (*ca*. >20 m apart) beneath the overstory trees of each forest. At each of the four locations, four samples were collected (>5 m apart) and composited into a single sample. The soil samples (0–10 cm depth) were collected in September of 2016 and October 2017, and the soil samples (20–30 cm depth) were collected in August 2017. Each sample was divided into a sub-sample for water content analysis, and a sub-sample for isotopic analysis of N. Soil water content was determined after samples were dried at 105°C for >3 days.

### DNA Extraction From Roots and Amplicon Sequencing

Root samples were carefully washed three times, and 300 root tips were picked up from each root sample and crushed in each 1.5 mL microtube using BioMasher (Nippi. Inc., Tokyo, Japan). To the crushed tissue, 350 μL of CTAB buffer (100 mM Tris; 1.4 M NaCl; 20 mM EDTA; 2% acetyl trimethyl ammonium bromide) was added, and the mixture was incubated for 30 min at 65°C (Sato and Murakami, 2008). After incubation, an equal volume of chloroform was added and centrifuged for 15 min at 12,000 rpm. The supernatant was then transferred to a new microtube. An equal volume of isopropanol and a one-tenth volume of sodium acetate (pH 5) were added to the supernatant and then the mixture was centrifuged for 5 min at 12,000 rpm. After being washed with 70% ethanol, the precipitated DNA was dissolved in 200 μL of TE buffer (10 mM Tris; 1 mM EDTA, pH 8.0). The DNA extracts were kept in a −20°C freezer until sequencing analysis.

We amplified the small subunit of the nuclear ribosomal RNA (SSU rDNA) in the DNA extracts using the primer sets AMV4.5NF/AMDGR (Sato et al., 2005) to reveal AM fungal community in the tree roots. Because all the understory trees are primarily considered as AM trees, AM fungal community was measured. The reaction solution ratio was 20:2:2:3:13 Q5 High-Fidelity DNA Polymerase (New England Biolabs Inc., MA, United States)/10 mM forward primer/10 mM reverse primer/10-fold-diluted DNA sample/sterilized distilled water. Cycling conditions were as follows: initial denaturation at 98°C for 30 s, followed by 35 cycles of 98°C for 10 s, 58°C for 30 s, and 72°C for 30 s, followed by a 2-min final extension at 72°C. Amplification was performed using two replicates from each sample, after which amplification was checked by agarose gel electrophoresis and the replicates were composited to make one solution for purification. Purification was conducted using Agencourt AMPure XP (Beckman Coulter Inc., Brea, CA, United States) following the manufacturer’s protocol. After purification, appropriate amounts of samples were combined into one tube and mixed to equalize the DNA quantity in each sample, which was computed using the Qubit dsDNA HS Assay Kit (Thermo Fisher Scientific). The combined samples were separated by the agarose gel electrophoresis, and the gels containing SSU rDNA genes were extracted by QIAquick Gel Extraction Kit (Qiagen, Hilden, Germany) following manufacture’s instruction. Based on the size and quality of DNA in the gel extracts, which were checked using the Agilent High Sensitivity DNA Kit and Agilent 2100 Bioanalyzer (Agilent Technologies, Santa Clara, CA, United States), a dilution library of approximately 25 pM was prepared. Using 25 μL of the targeted dilution library, emulsion PCR was conducted using the Ion PGM OT2 400 Kit (Thermo Fisher Scientific) according to the manufacturer’s instructions. After recovery of the ion spheres and enrichment, samples were loaded onto an Ion 318 Chip V2 instrument for sequencing using an Ion Personal Genome Machine (PGM) (Thermo Fisher Scientific). After analysis, sequence data with low-quality sequences and polyclonal sequences removed were exported as a FASTQ file from the PGM software. The barcode primer sequence was removed from the sequences, and the SSU rDNA sequences shorter than 250 bp in length were removed, then low-qualify sequences were discarded using a quality-filter of a maximum number of expected errors (E_max) of 0.5 using the FASTX toolkit. Sequences were dereplicated, singletons were removed, and clustering of OTUs at 97% similarity was conducted using USEARCH (Edgar, 2010). Sequences identified as chimeric were eliminated using UCHIME (Edgar et al., 2011), and the taxonomy was assigned with using the Maarj*AM* database (Öpik et al., 2010) and RDP algorithm at an 80% confidence threshold. These computer analyses were conducted using Quantitative Insights Into Microbial Ecology (QIIME) version 1.8.0 (Caporaso et al., 2011). All the OTUs were checked by the BLASTN program, then the OTUs that were not assigned as AM fungi were discarded. Sequence data were deposited in the Sequence Read Archive at NCBI under accession number DRA009209.

OTUs whose relative abundance was <1% were removed. The OTU tables were converted into 1 or 0 binary OTU tables, as >0 and 0 were defined “presence (1)” and “not presence (0),” respectively. AM fungi was categorized by the family level. The frequency was calculated as how many individuals had each AM family. Each understory tree species beneath the overstory trees of each forest have 5 replicates, so the range of frequency is from 0 to 5. We note that the surface of plant roots was carefully washed to avoid the contamination of soils so that the root fungal community could be clearly different from the soil fungal community (Supplementary Figure 3). Soil DNA was extracted from 0.25-g soil samples (0–10 cm depth) using the MoBio Powersoil DNA Isolation Kit (Mo Bio, Carlsbad, CA, United States).

### Measurement of the δ^15^N and δ^18^O and IER-Captured Nitrate Content

Leaf and root samples were washed with distilled water. The leaf, root, and bulk soil samples were oven-dried at 60°C for over 24 h and homogenized into a ground powder. A total of 10 g of bulk soil samples were hydrolyzed using *ca*. 20 ml of 1 M HCl to remove carbonate. Leaf and root samples were loaded into tin capsules for isotope analysis, and total C and N concentration and stable isotope ratios were measured using an isotope ratio mass spectrometer (DELTA V Advantage, Thermo Fisher Scientific Inc., United States) with an elemental analyzer (Flash 2000, Thermo Fisher Scientific Inc., United States). The N stable isotope ratios of bulk soil samples were analyzed with an Elemental Analyser (Eurovector) coupled to an Isotope Ratio Mass Spectrometer (Delta Plus XP, Thermo Fisher Scientific, United States). The precision of the on-line procedure was better than ±0.2‰ for δ^15^N.

Soil samples for extractable N analysis were extracted with 2 M KCl solution (soil: KCl = 1: 10, w/w). IERs were shaken with 20 ml of 2 M KCL for 20 min three times, making a total extract of 60 ml per one resin. Before preparing 2 M KCl solution, the KCl was muffled at 450°C for 4 h. The extracts were filtered using a precombusted glass-fiber filter (GF/F; Whatman Int. Ltd., Maidstone, United Kingdom, muffled at 450°C for 4 h). The KCl extracts were frozen until analyses of N concentration and isotopic composition. We measured the concentration of ammonium and nitrate in soil and resin extracts, and that of total extractable N in soil extracts, using a microplate reader (Synergy^TM^ HTX, BioTek, United States). For determining the concentration of ammonium and nitrate, the indophenol-blue method and a modified acidic Griess reaction (Miranda et al., 2001) were used, respectively. Total extractable N was oxidized to nitrate, using persulfate oxidization and measured as nitrate (Miyajima et al., 2005). The extractable organic N (EON) is then calculated as [EON] = [Total extractable N] − [Nitrate N] − [Ammonium N]. When [Nitrate N] was below the detection limit, we assigned [Nitrate N] as zero.

The δ^15^N and δ^18^O stable isotopes of nitrate were measured using the denitrifier method (Sigman et al., 2001; Casciotti et al., 2002; Thuan et al., 2018). We converted the nitrate into nitrous oxide (N_2_O) using a denitrifier (*Pseudomonas aureofacien*) that lacked the enzyme to convert N_2_O to N_2_. The produced N_2_O was introduced into the Isotope Ratio Mass Spectrometer (Sercon 20/22 equipped with Cryoprep and GC; Sercon Ltd., United Kingdom). The δ^15^N of total extractable N was determined using persulfate oxidation followed by the denitrifier method (Houlton et al., 2007; Koba et al., 2012). The ammonium in the KCl extract was recovered with the diffusion method (Holmes et al., 1998) and the ammonium was captured onto a glass fiber filter (muffled at 450°C for 4 h, GF/D; Whatman Int. Ltd., Maidstone, United Kingdom) during shaking for over 48 h and drying for over 24 h. The captured ammonium was converted into nitrate by persulfate oxidization, then its N isotope ratio was determined by the denitrifier method (Thuan et al., 2018).

The natural abundance of ^15^N and ^18^O was expressed in per mil (‰) deviation from international standards: δ^15^N or δ^18^O = [*R*_sample_/*R*_standard_−1] × 1000, where R is ^15^N/^14^N or ^18^O/^16^O, respectively. Atmospheric N and Vienna standard mean ocean water were used as the international standards for N and O, respectively. Calibrations for these isotopic analyses were carried out using several international and in-house standards [USGS32, 34, 35 and IAEA NO_3_^–^ for nitrate, USGS25, 26 and IAEA N-2 for ammonium and calibrated alanine, glycine, and histidine for total extractable N (Takebayashi et al., 2010)]. Natural abundance δ^15^N of soil nitrate was measured for nine samples collected from beneath black locust trees, and due to very low nitrate concentration, for only one sample beneath oak trees. For these 10 samples, δ^15^N_EON_ was calculated assigning [Nitrate] as zero. The δ^15^N of EON was calculated using the following mass and isotopic balance equation: δ^15^N_EON_ = {δ^15^N_T__otal extractable N_ × [Total extractable N] − (δ^15^N_nitrate_ × [Nitrate N] + δ^15^N_ammonium_ × [Ammonium N])}/[EON]. The difference in the δ^15^N between leaves and roots of the same individual tree was defined as δ^15^N_root–leaf_ = δ^15^N_root_ – δ^15^N_leaf_.

### Statistical Analysis

Non-metric multidimensional scaling (NMDS) analysis of AM fungal community structure dissimilarity based on the Bray–Curtis index was performed using the metaMDS function in the vegan package (Oksanen et al., 2016) of R software. The envfit function in the vegan package was used to identify significant correlations between the NMDS values of points and the species of the trees (one tree species = 1, other tree species = 0), and the presence of fungal families and OTUs, to illustrate the vectors on the NMDS ordination plot. To determine the effects of soil environment, the extractable N content, moisture, and pH in soil around each tree individual were illustrated on the ordination. To identify the specific AM fungi that contribute greatly to the N acquisition of the host tree, the vectors of δ^15^N_root–leaf_ (the indicator of mycorrhizal dependence) and leaf N concentration of the host trees were tried to be illustrated on the ordination. Permutational multivariate analysis of variance (PerMANOVA) was performed to test the significance of the effect of overstory tree type and the host tree species on the microbial community of understory trees, using the adonis function in the R vegan package. The 1 or 0 binary OTU tables were used to illustrate NMDS and conduct PerMANOVA. Because three of the oak trees had no AM fungi, and the other oak trees had only one or two AM fungi, oak trees were not included in the NMDS analysis.

We used two-way analysis of variance (ANOVA) to test for significant differences between overstory tree type (black locust [non-ECM] or oak [ECM]) and plant species for the following: the total number of AM fungal OTUs in roots, the leaf N concentration, and the content, δ^15^N and/or δ^18^O, of N in soils and captured in IER. We set the difference of sampling occasion (timing) as a random effect. We used generalized mixed model (GLMM) using the binomial family to test for significant differences between overstory tree type and plant species for the presence (1 or 0) of AM family and OTUs in roots. Tukey’s multiple comparisons were used to examine potential differences among δ^15^N of leaves, roots, and soils. We used a paired *t*-test to examine potential differences in δ^15^N between the leaf and root of the same individual plants. Student’s *t*-test was used to test for significant differences in leaf N concentration between the same plant species across beneath the different types of overstory trees. Prior to these tests except for GLMM, we tested for normality and homogeneity of variance in the data using the Kolmogorov–Smirnov and median Levene’s tests, respectively. The car package in R software (version 3.1.2; R Core Team, 2014) was used to perform Levene’s test (Fox et al., 2014). When the *P*-values of Kolmogorov–Smirnov or Levene’s test prior to ANOVA was <0.05, the data were square-root or reciprocally transformed. When the *P*-values of Kolmogorov–Smirnov or Levene’s test prior to *t*-test was <0.05, Welch’s *t*-test was alternatively used. When the *P*-values of Kolmogorov–Smirnov test was >0.05 but the *P*-values of Levene’s test were <0.05 prior to the multiple comparisons, Games–Howell’s test was alternatively used. We chose a significance level of *P* < 0.05 for all tests.

## Results

### AM Fungal Community in the Tree Roots

The total read number we obtained from root samples was 698,455 (the average ± SD was 9,978 ± 3,923 reads per sample). The total read number that was confirmed as AM fungi by the BLASTN was 63,243 (903 ± 558 reads per sample). The total number of AM fungal OTUs was 174, and beneath the black locust trees was 109 OTUs, and beneath the oak trees 91 OTUs. The average ± SD of the number of OTUs was 12 ± 9 per sample.

**FIGURE 1:**
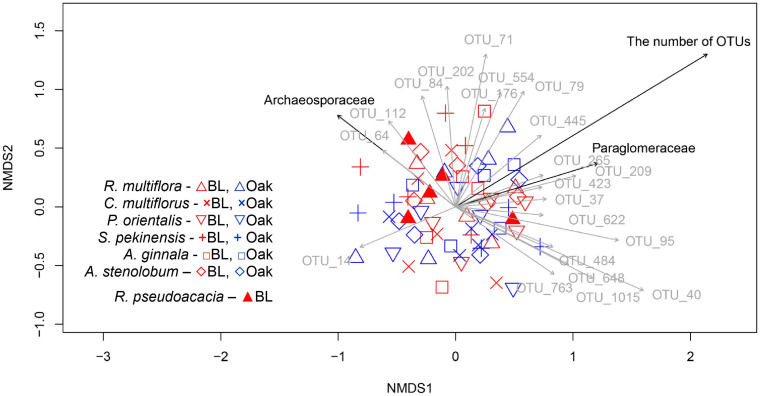
Non-metric multidimensional scaling (NMDS) based on root AM fungal community dissimilarities. Red and blue symbols represent understory trees beneath black locust and oak trees, respectively. The symbols indicate as follows: Δ; *Rosa multiflora*, ×; *Cotoneaster multiflorus*, ∇; *Platycladus orientalis*, +; *Syringa pekinensis*, □; *Acer ginnala*, ⋄; *Acer stenolobum*, ▲; *Robinia pseudoacacia*. The stress value was 0.27. Only significant vectors are plotted on the ordination. Black vectors represent the presence of fungal family and the total number of operational taxonomic units (OTUs). Dark gray vectors represent the presence of frequent OTUs. The experimental design (overstory tree type and host tree species) and soil chemical properties were not plotted.

The AM fungal community structure was not significantly affected by overstory tree type (*F* = 1.51, *R*^2^ = 0.03, *P* = 0.123), by the host plant species (*F* = 0.893, *R*^2^ = 0.07, *P* = 0.696), or by the interaction between overstory tree type and understory tree species (*F* = 1.126 *R*^2^ = 0.09, *P* = 0.259) based on PerMANOVA. NMDS also did not show a difference in the mycorrhizal community composition between beneath the black locust and oak trees (Figure 1). The community composition associated with understory trees overlapped with each other. However, when comparing samples from beneath non-ECM and ECM trees to test these differences for each individual species, the significant effect of the overstory tree type was only present in *C. multiflorus* (Supplementary Figure 3). The overstory tree type, host tree species, extractable N content, and pH in the soil associated with the trees (Supplementary Figure 1) were not significantly correlated with the community composition (Figure 1).

**FIGURE 2:**
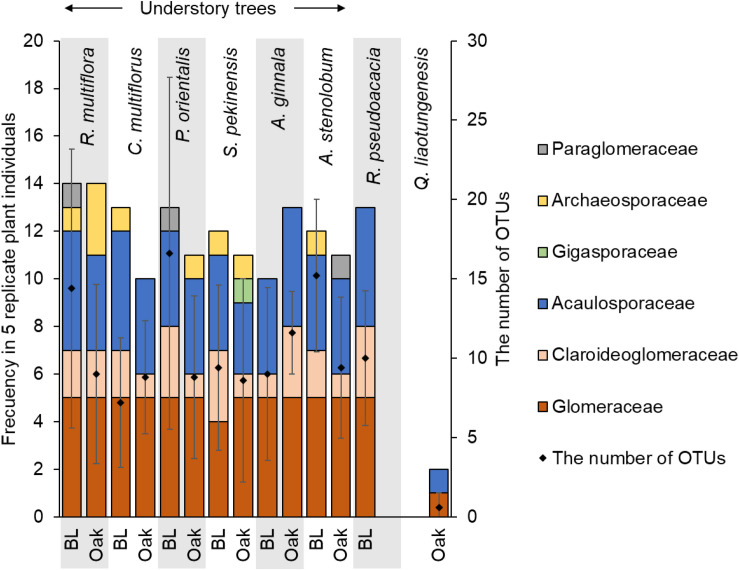
The frequency of AM fungal family and the averaged total number of AM fungal OTUs in 5 replicate plant individual roots collected from beneath black locust (BL) and oak trees. There were no significant effects of overstory tree type and host tree species based on the GLMM (for the frequency) and the two-way ANOVA (for the number of OTUs) with the overstory type and species. The overstory tree samples were not included in the GLMM and two-way ANOVA comparison. Red group belongs to the order Glomerales.

The most frequent family of AM fungi was Glomeraceae followed by Acaulosporaceae (Figure 2). The presence of all the families was not significantly affected by the overstory tree type or the host tree species (Figure 2 and Supplementary Figure 4). The number of OTUs was also not significantly affected by the overstory tree type or the host tree species. Some of AM fungal OTUs, OTU 37, 544, 265, 622 (Glomeraceae) and OTU 112 (Claroideoglomeraceae), more frequently appeared beneath non-ECM (black locust) trees than beneath ECM (oak) trees (Supplementary Figure 4).

### The Chemical Properties of the Understory Trees and Soils

**FIGURE 3:**
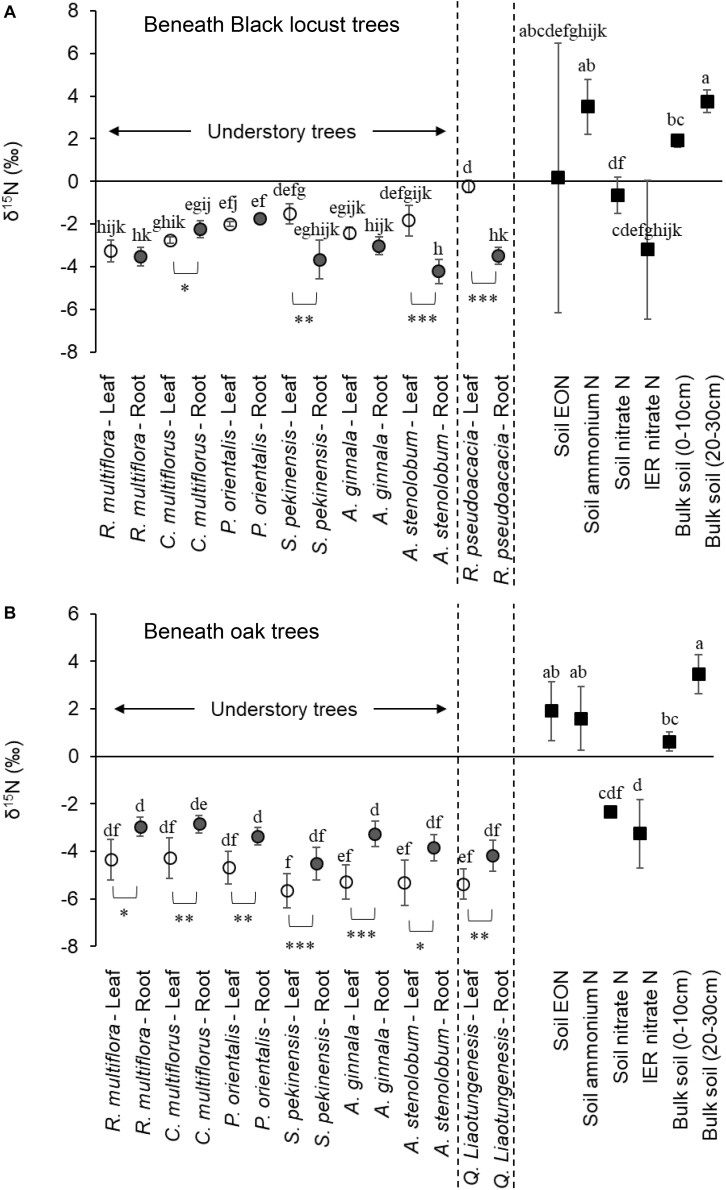
The δ^15^N of leaves and roots of the six understory trees and the overstory trees, and the δ^15^N of soil N sources (soil extractable N and bulk soil) beneath **(A)** black locust and **(B)** oak trees (mean ± SD). The results of the paired *t-*test between δ^15^N of leaves and roots from the same individual trees were shown below of the plots. ^∗^*P* < 0.05, ^∗∗^*P* < 0.01, ^∗∗∗^*P* < 0.001. Different lowercase letters above the plots indicate significant pairwise differences (Tukey–Kramer or Games–Howell test).

**FIGURE 4:**
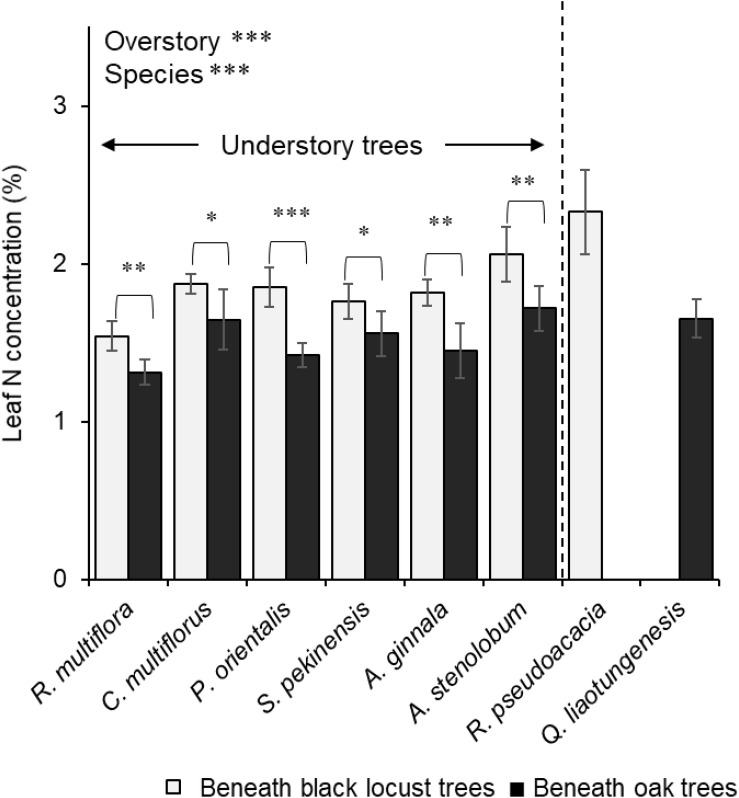
Leaf N concentrations of the six understory trees and the overstory trees beneath the black locust overstory and oak overstory trees. Values are means ± S.D. Significant differences between different overstory tree types in each species (identified by Student’s or Welch’s *T*-test) are indicated on the bar. The results of two-way ANOVA with the overstory tree type (Overstory) and the tree species (Species) are shown at the top. The overstory tree samples were not included into the two-way ANOVA comparison. ^∗^*P* < 0.05, ^∗∗^*P* < 0.01, ^∗∗∗^*P* < 0.001.

Beneath oak trees, leaf δ^15^N was consistently lower than root δ^15^N in all understory tree species (Figure 3B). On the other hand, beneath black locust trees, the relationship between leaf and root δ^15^N varied and depended on the understory tree species (Figure 3A). Leaf δ^15^N of *S. pekinensis* and *A. stenolobum* was significantly higher than their root δ^15^N, although leaf δ^15^N of *C. multiflorus* was lower than its root δ^15^N beneath black locust trees (Figure 3A). Leaf N concentration was significantly affected by the overstory tree type, and consistently higher beneath black locust than the oak trees in all understory tree species (Figure 4).

Beneath black locust trees, the δ^15^N of understory trees was not significantly different from the δ^15^N of soil EON, IER-captured nitrate, and that of *P. orientalis* and leaves of *S. pekinensis* and *A. stenolobum* was also not significantly different from that of soil nitrate (Figure 3A). However, the δ^15^N of understory trees was consistently lower than that of soil ammonium beneath black locust trees (Figure 3A). The δ^18^O of nitrate in soils and captured in IER beneath black locust trees was lower than 10‰ (Table 1). Beneath oak trees, the δ^15^N of the leaves and roots of understory trees were significantly lower than δ^15^N of EON and ammonium (Figure 3B). The δ^15^N of soil nitrate beneath oak trees was measured only in one sample (Table 1). The δ^15^N of IER-captured nitrate was not significantly different with the δ^15^N of some understory trees (Figure 3B), and δ^18^O of nitrate captured in IER was much higher than 10‰ beneath oak trees (Table 1).

**TABLE 1:** The content and δ^15^N of soil extractable N in soil (0–10 cm depth), nitrate N in ion-exchanged resin (IER) and bulk soil (0–10 cm depth and 20–30 cm depth), and the δ^18^O of nitrate N in soil (0–10 cm depth) and captured in IER (mean ± SD).

		Black locust trees	(*n*)	Oak trees	(*n*)	*F*-value
Content (mg kg^–1^) or (mg cap^–1^)	Soil EON	18.6 ± 10.8	(36)	39.5 ± 18.9	(36)	59.3 ***
	Soil ammonium N	9.1 ± 3.3	(36)	12.3 ± 3.3	(36)	66.5 ***
	Soil nitrate N	7.2 ± 3.5	(36)	0.7 ± 0.6	(36)	238.4 ***
	IER nitrate N	0.24 ± 0.18	(7)	0.03 ± 0.02	(7)	18.1 **
	Soil total N (0–10 cm depth)	2312 ± 434	(36)	3234 ± 571	(36)	68.4 ***
	Soil total N (20–30 cm depth)	544 ± 44	(4)	629 ± 76	(4)	3.8
δ^15^N (‰)	Soil EON	0.2 ± 6.3	(10)	1.9 ± 1.2	(10)	0.7
	Soil ammonium N	3.5 ± 1.3	(10)	1.6 ± 1.3	(10)	10.1 **
	Soil nitrate N	−0.7 ± 0.9	(9)	−2.4	(1)	3.8
	IER nitrate N	−3.2 ± 3.2	(7)	−3.3 ± 1.4	(7)	0.0
	Soil total N (0–10 cm depth)	1.9 ± 0.3	(8)	0.6 ± 0.4	(8)	54.4 ***
	Soil total N (20–30 cm depth)	3.8 ± 0.5	(4)	3.5 ± 0.8	(4)	0.4
δ^18^O (‰)	Soil nitrate N	1.5 ± 2.3	(9)	−3.1	(1)	9.8 *
	IER nitrate N	4.1 ± 2.3	(7)	38.7 ± 16.2	(7)	31.6 ***

(n) shows the number of samples. Right side shows F-values and P-values (*P < 0.05, **P < 0.01, ***P < 0.001) based on one-way ANOVA with the overstory tree type (beneath black locust or oak trees). The models were performed with sampling occasions as a random variable. The data of soil extractable N content and total N content was from Tatsumi et al. (2019, 2020).

In the surface soil (0–10 cm depth), EON and soil ammonium content were not significantly different between different types of overstory trees, but the soil nitrate content was 10 times and significantly higher beneath the black locust than beneath oak trees (Table 1, Tatsumi et al., 2020). The content of nitrate captured in IER was higher beneath black locust than oak trees (Table 1). The δ^15^N of bulk surface soil and soil ammonium was also significantly higher beneath black locust than beneath oak trees (Table 1).

## Discussion

In contrast to our expectation in the first hypothesis, the mycorrhizal community composition associated with roots of understory trees was not significantly different between beneath different types of overstory trees (Figure 1). However, some OTUs, especially in Glomeraceae, were more frequent beneath the non-ECM (black locust) trees than the ECM (oak) trees, and the community composition of *C. multiflorus* was significantly different between beneath the non-ECM and ECM trees (Figure 1 and Supplementary Figure 3). The higher frequency of some OTUs in Glomeraceae beneath the non-ECM trees may have been caused by soil N availability, as Glomeraceae is reported to increase under high soil N availability (Treseder and Allen, 2002; Egerton-Warburton et al., 2007), and soil N availability was higher beneath non-ECM than ECM trees (Table 1).

In agreement with the second hypothesis that the understory trees beneath the non-ECM overstory trees access nitrate, δ^15^N of soil nitrate (especially, IER-captured nitrate) was similar to δ^15^N of the leaves of the understory trees (Figure 3A). Further, the understory trees beneath the non-ECM overstory trees did not depend strongly upon mycorrhizal fungi, as shown by the leaf δ^15^N not being significantly lower than root δ^15^N, except for *C. multiflorus* (Figure 3A). The fact that δ^15^N of ammonium was higher than δ^15^N of the understory trees suggests that the understory trees likely did not use ammonium (Figure 3A). Although the δ^15^N of soil EON was also not significantly different from the δ^15^N of the understory trees, EON might not be major N source because most of the EON is high-molecular-weight and recalcitrant (Jones et al., 2005), and an only small part of EON is possible N source for plants (Takebayashi et al., 2010). Supporting the idea that the understory trees take up nitrate, the δ^15^N of leaves was higher than that of roots, especially of *S. pekinensis* and *A. stenolobum*. Nitrate assimilation occurs both in roots and shoots, with lighter nitrate typically assimilated in roots, and then the heavier nitrate is typically transferred to shoots and leaves, leading to greater leaf δ^15^N than root δ^15^N (Yoneyama and Kaneko, 1989; Evans et al., 1996; Evans, 2001). Further, the active nitrification beneath the non-ECM overstory trees could support the use nitrate by the understory trees. The δ^18^O of soil and IER-captured was <10‰ beneath the non-ECM overstory trees, suggesting that nitrate comes primarily from nitrification, although the IER-captured nitrate beneath the ECM overstory trees was likely derived mostly from atmosphere (Table 1; Kendall et al., 2007; Fang et al., 2012). Also, the relatively greater δ^15^N values in soils beneath the non-ECM than ECM trees support the idea that nitrification rate is higher beneath the non-ECM overstory trees since they are typically positively correlated (Templer et al., 2007), although the soil δ^15^N values might be caused by the probably greater δ^15^N values of litter input under the non-ECM than ECM overstory trees (Figure 3A). Thus, the understory trees beneath the non-ECM overstory trees likely take up a rich nitrate pool, which is derived from active nitrification, being consistent with our second hypothesis. However, it is still not clear from our experimental design, which trait of black locust, AM species or N-fixers presence/activity, caused the rich nitrate pool.

In support of our third hypothesis, all plant species beneath the ECM overstory trees likely took up N via mycorrhizal fungi, as indicated by the fact that leaf δ^15^N was lower than root δ^15^N (Figure 3B), which suggests mycorrhizal fungi in roots transferred lighter N to leaves (Kohzu et al., 2000; Emmerton et al., 2001; Hobbie and Colpaert, 2003). We could not separate the roots and mycorrhizal fungi for isotope analysis, so the high root δ^15^N may have come from the N remaining in the fungal root component (Schweiger, 2016). Although the transfer of ^15^N-depleted N from AM fungi to host plants has not clearly been demonstrated compared to ECM fungi (Aguilar et al., 1998; Wheeler et al., 2000), AM plants are more ^15^N-depleted than non-mycorrhizal plants (Craine et al., 2015; Schweiger, 2016), or rarely as much ^15^N-depleted as co-occurring ECM plants, which may indicate AM fungi also fractionate against ^15^N (Hobbie and Hobbie, 2008). Besides, a study using calculation models and field sampling suggested there is a 2–4‰ fractionation of N in AM transfer to plants (Jach-Smith and Jackson, 2020), and another study also supported the fractionation of AM fungi based on the fact that leaf ^15^N depletion coincided with root ^15^N enrichment (Schweiger, 2016). In contrast, it is still possible that understory trees beneath the ECM overstory trees directly take up nitrate without mycorrhizal association as indicated by δ^15^N of soil nitrate being similar to δ^15^N of the leaves of the understory trees, and that the internal fractionation within plants is considered to cause 2‰ difference in δ^15^N between leaf and root (Dawson et al., 2002; Houlton et al., 2007). However, the slow nitrification process beneath the ECM overstory trees, demonstrated by our previous study (Tatsumi et al., 2020) and the total soil δ^15^N and δ^18^O values of nitrate as mentioned above, suggest that direct uptake without mycorrhizal fungi was not major N source for understory trees. Besides, the internal fractionation cannot explain why the consistently lower leaf than root δ^15^N was only observed beneath the ECM overstory trees. Thus, the understory trees beneath the ECM overstory trees likely more depended upon mycorrhizal fungi for N acquisition, following our third hypothesis. As AM fungal community composition was similar between beneath the non-ECM and ECM trees, the dependence on the same AM fungi should largely change between beneath the non-ECM and ECM trees. However, we did not measure rates of root colonization, so more detailed research is needed to determine the mycorrhizal dependency of plants in these dry forests.

We also found that the patterns of N uptake of the understory trees were affected by the understory tree species. For example, *C. multiflorus* had significantly lower δ^15^N in leaves than roots beneath the non-ECM overstory trees, while other understory tree species had higher or similar δ^15^N in leaves than roots (Figure 3A). The Genus *Cotoneaster* was reported to show lower growth when fertilized with nitrate than with ammonium (Kraus et al., 2002). *C. multiflorus* may not actively access the rich nitrate pool beneath the non-ECM overstory trees.

We conclude that while tree species is an important factor for describing patterns of N uptake by understory trees, we found that differences in understory N utilization also depends on the type of overstory trees present. We suggest that the N uptake patterns of co-existing understory trees vary beneath different types of overstory trees, although it is necessary to compare other non-ECM and ECM overstory tree species. With greater sampling, we may be able to separate the effects of mycorrhizal type vs. the effects of the presence of N fixers on the ability for understory trees to take up N. However, black locust and oak are the most common species in this region, so our findings should be at least important for understanding the reforestation and succession process in this region. Furthermore, the higher N concentrations of understory trees beneath the non-ECM than ECM overstory trees (Figure 4) may contribute to further acceleration of N cycling as a positive feedback, because high N concentrations of litter increase rates of decomposition (Cotrufo et al., 1995; Sun et al., 2018), although more research about N in litterfall of understory trees is needed.

## Data Availability Statement

The datasets generated for this study can be found in online repositories. The names of the repository/repositories and accession number(s) can be found in the article/Supplementary Material.

## Author Contributions

CT and RT designed the research. CT, FH, TT, SD, NY, and RT conducted the fieldwork. CT, FH, TT, WS, KK, and KF conducted the laboratory work. CT, FH, TT, KK, PT, and RT critically contributed to interpreting the data. CT and RT led the writing of the manuscript. All authors reviewed the manuscript and gave final approval for publication.

## Conflict of Interest

The authors declare that the research was conducted in the absence of any commercial or financial relationships that could be construed as a potential conflict of interest.
