# Supplementary material for: Arbuscular Mycorrhizal Community in Roots and Nitrogen Uptake Patterns of Understory Trees Beneath Ectomycorrhizal and Non-ectomycorrhizal Overstory Trees
Source: Front Plant Sci. 2021 Jan 14;11:583585. doi: 10.3389/fpls.2020.583585 (PMC7840530; doi:10.3389/fpls.2020.583585)
Supplement: Supplementary file 1 [file Data_Sheet_1.pdf]

(Title)

Arbuscular mycorrhizal community in roots and nitrogen uptake patterns of understory trees beneath ectomycorrhizal and non-ectomycorrhizal overstory trees

(Authors)

Chikae Tatsumi[1,2, 3],*, Fujio Hyodo[4], Takeshi Taniguchi[5], Weiyu Shi[6], Keisuke Koba[7], Keitaro Fukushima[7], Sheng Du[8], Norikazu Yamanaka[5], Pamela Templer[3], Ryunosuke Tateno[9]

1) Research Faculty of Agriculture, Hokkaido University, 060-8589, Japan

2) Graduate School of Agriculture, Kyoto University, Kyoto 606-8502, Japan

3) Department of Biology, Boston University, Boston, MA, 02215, USA

4) Research Core for Interdisciplinary Sciences, Okayama University, Okayama, 700-8530, Japan

5) Arid Land Research Center, Tottori University, Tottori 680-0001, Japan

6) School of Geographical Sciences, Southwest University, Chongqing 400715, China

7) Center for Ecological Research, Kyoto University, Shiga 520-2113, Japan

8) State Key Laboratory of Soil Erosion and Dryland Farming on Loess Plateau, Institute of Soil and Water Conservation, Chinese Academy of Sciences, Yangling 712100, China

9) Field Science Education and Research Center, Kyoto University, Kyoto 606-8502, Japan

(*Corresponding Author)

Chikae Tatsumi

Research Faculty of Agriculture, Hokkaido University, 060-8589, Japan

E-mail address: chikae@chem.agr.hokudai.ac.jp

Table S1

(a)

ID	Nodule	Root tips	Root length (cm)
1	0	114	11.5
2	0	90	15.7
3	0	249	17.1
4	0	91	12.9
5	0	346	18.7
6	0	272	17.8

(b)

	Beneath black locust trees	Beneath oak trees	F value	
0 - 10 cm soil	258.6 ± 30.4	302.6 ± 34.6	41.2	***
20 - 30 cm soil	323.3 ± 32.2	355.8 ± 27.4	2.4	

Fig. S1

(a)

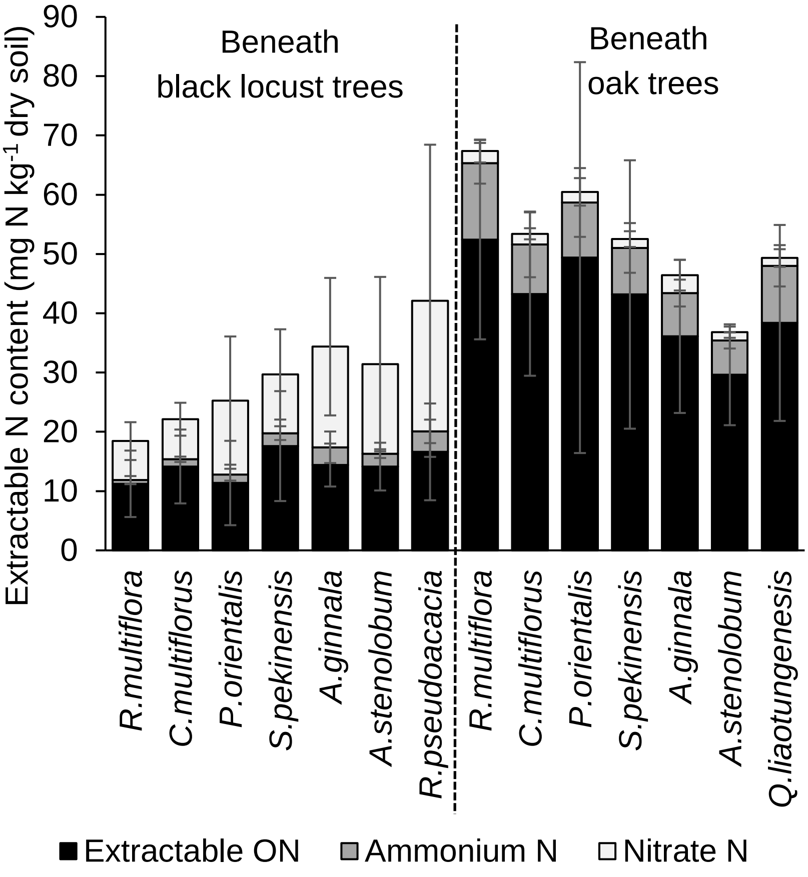
Extractable N content (mg N kg-1 dry soil)
0
10
20
30
40
50
60
70
80
90
Beneath black locust trees
Beneath oak trees
R.multiflora
C.multiflorus
P.orientalis
S.pekinensis
A.ginnala
A.stenolobum
R.pseudoacacia
R.multiflora
C.multiflorus
P.orientalis
S.pekinensis
A.ginnala
A.stenolobum
Q.liaotungenesis
Extractable ON
Ammonium N
Nitrate N

(b)

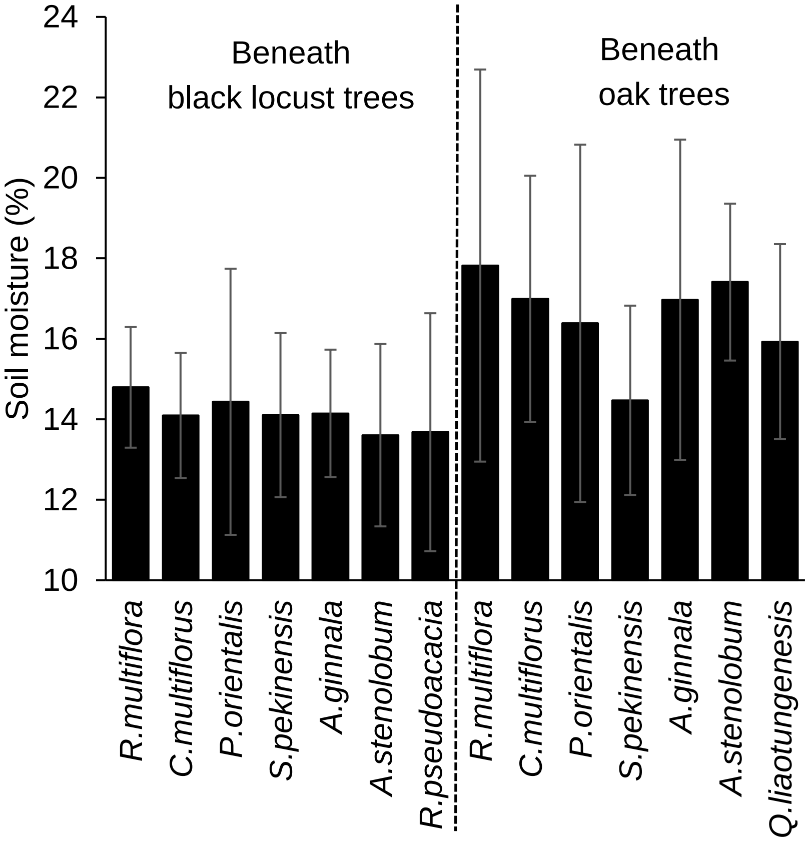
Soil moisture (%)
10
12
14
16
18
20
22
24
Beneath black locust trees
Beneath oak trees
R.multiflora
C.multiflorus
P.orientalis
S.pekinensis
A.ginnala
A.stenolobum
R.pseudoacacia
R.multiflora
C.multiflorus
P.orientalis
S.pekinensis
A.ginnala
A.stenolobum
Q.liaotungenesis

(c)

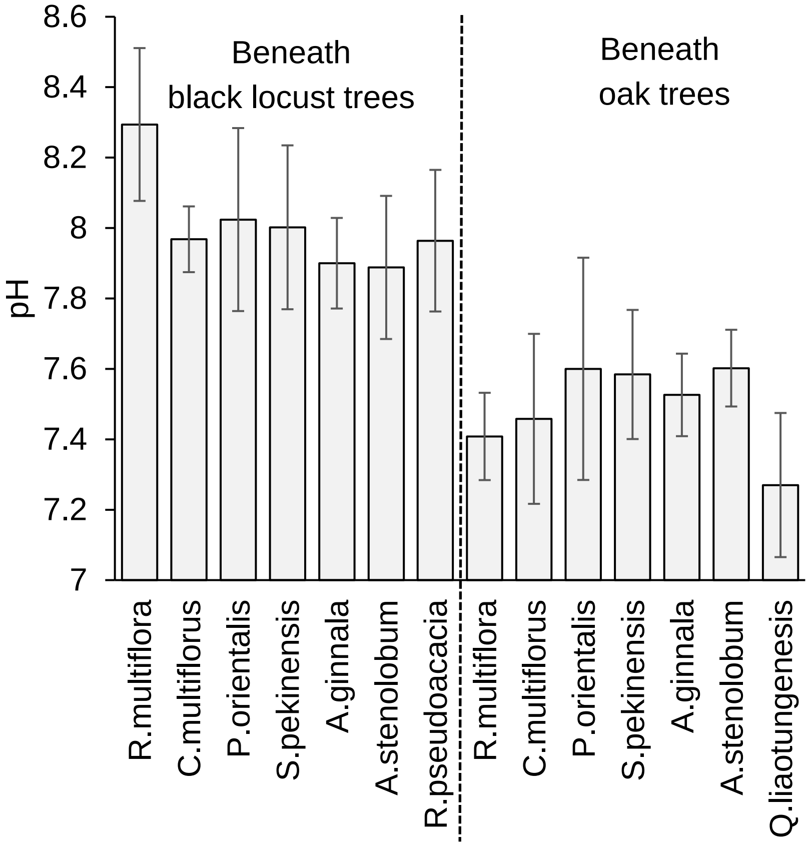
pH
8.6
8.4
8.2
8
7.8
7.6
7.4
7.2
7
Beneath
black locust trees
Beneath
oak trees
R.multiflora
C.multiflorus
P.orientalis
S.pekinensis
A.ginnala
A.stenolobum
R.pseudoacacia
R.multiflora
C.multiflorus
P.orientalis
S.pekinensis
A.ginnala
A.stenolobum
Q.liaotungenesis

Fig. S2

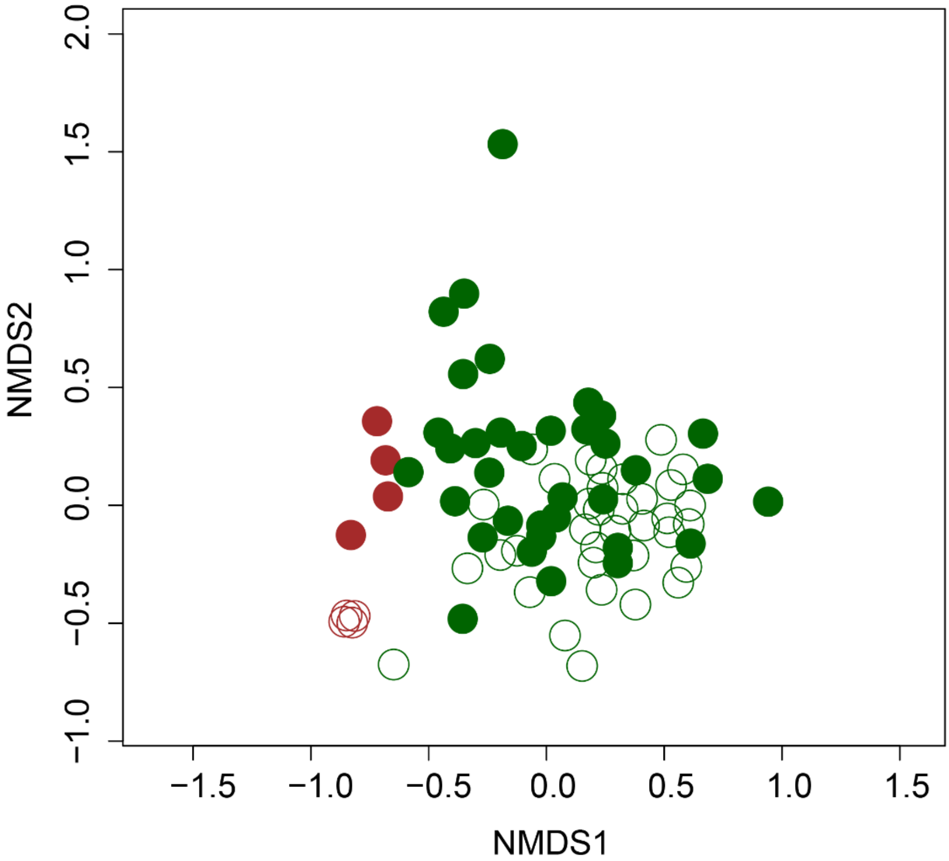

Fig. S3

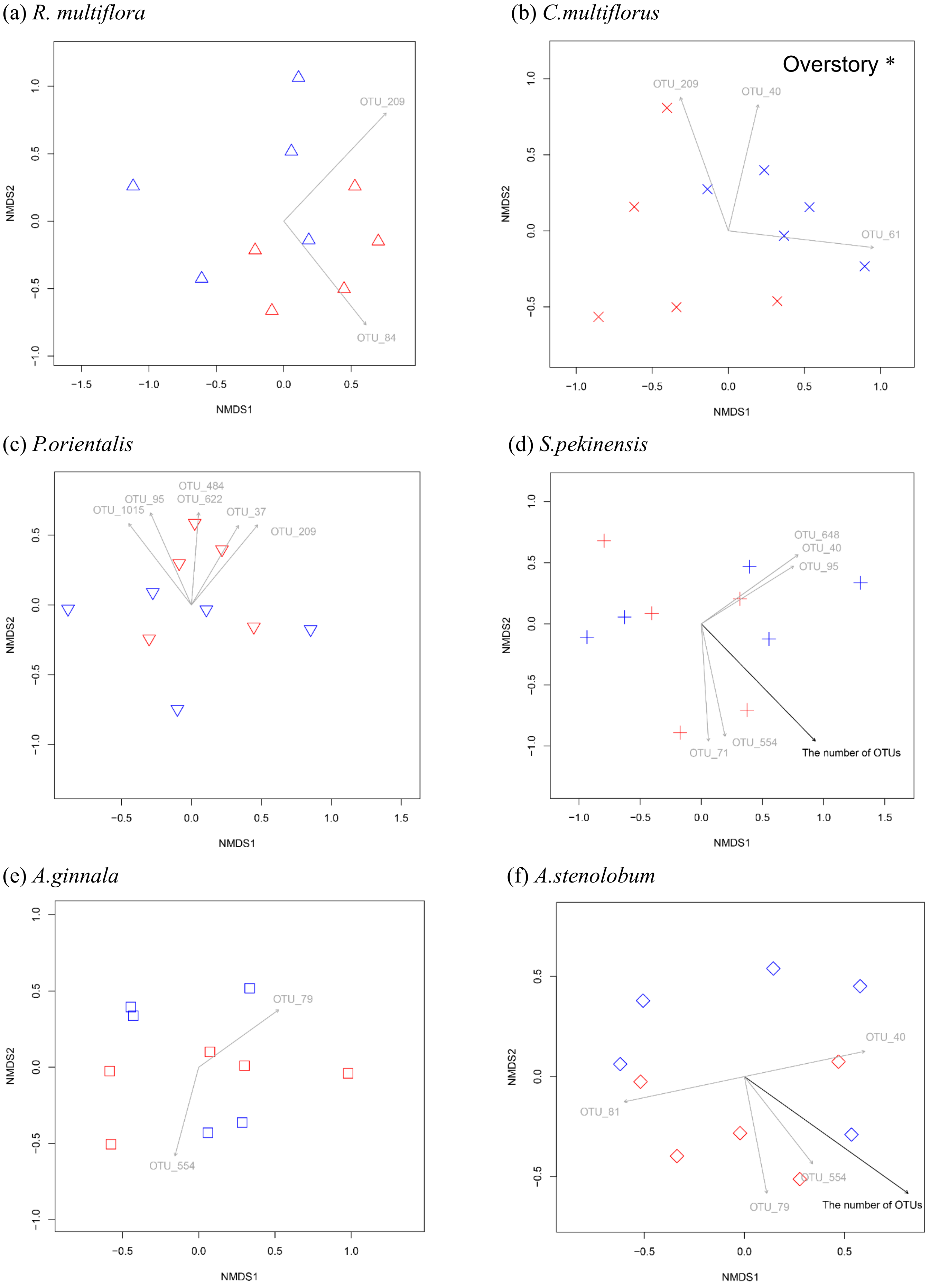
(a) R. multiflora
OTU_209
OTU_84
NMDS1
NMDS2
(b) C.multiflorus
Overstory *
OTU_209
OTU_40
OTU_61
NMDS1
NMDS2
(c) P.orientalis
OTU_484
OTU_95
OTU_622
OTU_1015
OTU_37
OTU_209
NMDS1
NMDS2
(d) S.pekinensis
OTU_648
OTU_40
OTU_95
OTU_554
OTU_71
The number of OTUs
NMDS1
NMDS2
(e) A.ginnala
OTU_79
OTU_554
NMDS1
NMDS2
(f) A.stenolobum
OTU_40
OTU_81
OTU_554
OTU_79
The number of OTUs
NMDS1
NMDS2

Fig. S4

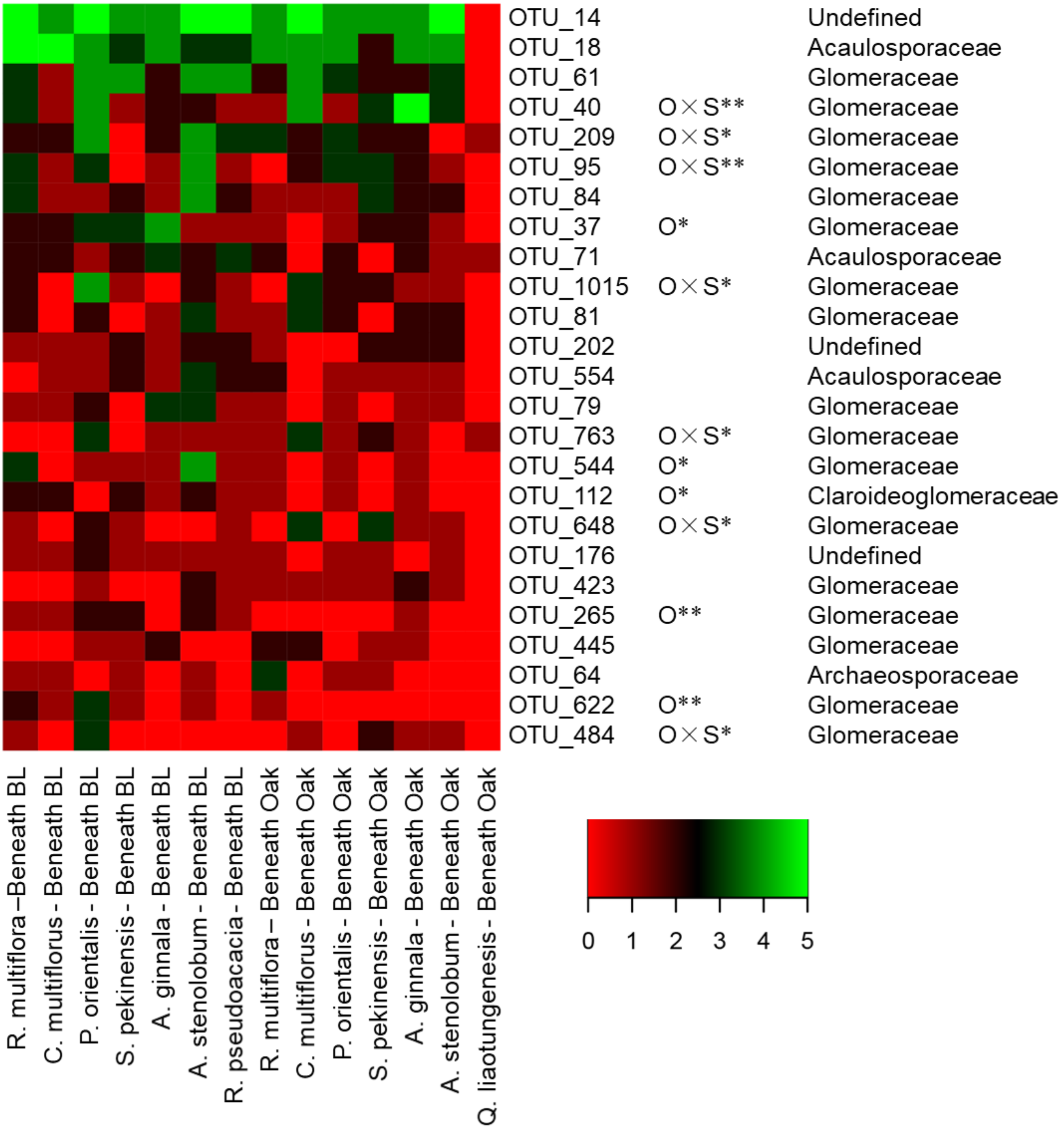
OTU_14 Undefined
OTU_18 Acaulosporaceae
OTU_61 Glomeraceae
OTU_40 O×S** Glomeraceae
OTU_209 O×S* Glomeraceae
OTU_95 O×S** Glomeraceae
OTU_84 Glomeraceae
OTU_37 O* Glomeraceae
OTU_71 Acaulosporaceae
OTU_1015 O×S* Glomeraceae
OTU_81 Glomeraceae
OTU_202 Undefined
OTU_554 Acaulosporaceae
OTU_79 Glomeraceae
OTU_763 O×S* Glomeraceae
OTU_544 O* Glomeraceae
OTU_112 O* Claroideoglomeraceae
OTU_648 O×S* Glomeraceae
OTU_176 Undefined
OTU_423 Glomeraceae
OTU_265 O** Glomeraceae
OTU_445 Glomeraceae
OTU_64 Archaeosporaceae
OTU_622 O** Glomeraceae
OTU_484 O×S* Glomeraceae
R. multiflora –Beneath BL
C. multiflorus - Beneath BL
P. orientalis - Beneath BL
S. pekinensis - Beneath BL
A. ginnala - Beneath BL
A. stenolobum - Beneath BL
R. pseudoacacia - Beneath BL
R. multiflora – Beneath Oak
C. multiflorus - Beneath Oak
P. orientalis - Beneath Oak
S. pekinensis - Beneath Oak
A. ginnala - Beneath Oak
A. stenolobum - Beneath Oak
Q. liaotungenesis - Beneath Oak
0 1 2 3 4 5

Table legends

Table S1 (a) The number of nodules, root tips, and main root length of black locust trees. 6 black locust individuals (ID 1 - 6) were randomly selected, and one of their roots growing in 0-10cm depth soil was sampled beneath black locust trees. The number of nodules, root tips were visually counted, and the length of the thickest and longest main roots was measured. (b) Predicted abundance of N-fixing gene (nitrogenase, EC1.18.6.1) in 1000 reads per samples enumerated using the Phylogenetic Investigation of Communities by Reconstruction of Unobserved States (PICRUSt) pipeline procedures for soils beneath black locust trees and oak trees. The method of soil sampling, DNA extraction, and sequencing analysis was described in Tatsumi et al (2020). Values are means ± SDs. The F values and P values ($P < 0.05$, ** $P < 0.01$, *** $P < 0.001$) were shown as the result of one-way ANOVA of the overstory tree type. The activities of N_2 fixers are limited under drought stress (Boring and Swank 1984; Polania et al. 2016, but see Wurzburger and Miniat 2014). Tateno et al. (2007) reported that the N accumulation in the soil of our site was much reduced in comparison with that in soils of black locust forests receiving more precipitation.

Figure legend

Fig. S1 (a) Extractable N content, (b) moisture and (c) pH in the soil around the understory and overstory tree individuals. Values are means ± SDs. Soil water content was measured by drying soil at 105°C for more than 3 days. Soil pH was measured using a pH meter (D-51, HORIBA, Kyoto, Japan) with a 2:5 soil/water suspension. Soil dissolved N were extracted with 2 M KCl at a 1:10 soil/extractant ratio. The amounts of total extractable N in the extracts were measured using TOC-LCPH/CPN+TNM-L (SHIMADZU, Kyoto, Japan) by 720 °C catalytic thermal decomposition/chemiluminescence methods. The amounts of ammonium and nitrate N in the extracts were measured using a Bran + Luebbe AutoAnalyzer III (BLTEC, Tokyo, Japan) with the colorimetric method. The amount of soil KCl-extractable organic N (EON) was calculated based on the differences between total extractable N and inorganic N, which was the sum of ammonium and nitrate N.

Fig. S2 Non-metric multidimensional scaling (NMDS) based on Bray-Curtis dissimilarities of fungal

community structures in roots and soils. The fill and color represent the overstory tree type and sample type (root or soil), respectively. Open and filled symbols indicate samples from beneath black locust trees and oak trees, respectively. Green and brown symbols refer to roots and soil, respectively. Non-metric multidimensional scaling (NMDS) analysis of community structure dissimilarity based on the Bray-Curtis index was performed using the metaMDS function in the vegan package (Oksanen and others 2016) of R.

Fig. S3 The NMDS based on root AM fungal community dissimilarities of each understory tree species beneath black locust and oak trees. Red and blue symbols represent the trees beneath the black locust and oak trees, respectively. Only significant vectors are plotted on the ordination. The upper left shows the significant effect of overstory tree type based on the one-way PerMANOVA.

Fig. S4 Relative frequency of AM fungal OTUs in roots of the co-existing understory trees beneath black locust (BL) trees and oak trees. The right side shows OTU ID, the result of GLMM, and the taxonomy (The Family). The significant effects of the overstory type (O), the tree species (S) and the interaction (Overstory×Species; O×S) were shown as the result of GLMM of the overstory type and the tree species. The overstory tree samples were not included into the GLMM. $P < 0.05$, ** $P < 0.01$, *** $P < 0.001$. OTUs which appeared in > 9 individuals were shown. OTUs were aligned from top to bottom along the total frequency in roots of 70 tree individuals.

Reference

Boring LR, Swank WT. 1984. Symbiotic nitrogen fixation in regenerating black locust (*Robinia pseudoacacia* L.) stands. For Sci 30: 528-537.

Oksanen AJ, Blanchet FG, Friendly M, Kindt R, Legendre P, Mcglinn D, Minchin PR, Hara RBO, Simpson GL, Solymos P, Stevens MHH, Szoecs E. 2016. Package 'vegan'(Version 2.4-0).

Polania J, Poschenrieder C, Rao I, Beebe S. 2016. Estimation of phenotypic variability in symbiotic nitrogen fixation ability of common bean under drought stress using ^{15}N natural abundance in grain. Eur J Agron

79: 66–73.

Tateno R, Tokuchi N, Yamanaka N, Du S, Otsuki K, Shimamura T, Xue Z, Wang S, Hou Q. 2007. Comparison of litterfall production and leaf litter decomposition between an exotic black locust plantation and an indigenous oak forest near Yan'an on the Loess Plateau, China. For Ecol Manage 241: 84–90.

Tatsumi C, Taniguchi T, Du S, Yamanaka N, Tateno R. 2020. Soil nitrogen cycling is determined by the competition between mycorrhiza and ammonia-oxidizing prokaryotes. Ecology. 101: e02963

Wurzburger N, Miniat CF. 2014. Drought enhances symbiotic dinitrogen fixation and competitive ability of a temperate forest tree. Oecologia 174: 1117–26.
